# RILP suppresses invasion of breast cancer cells by modulating the activity of RalA through interaction with RalGDS

**DOI:** 10.1038/cddis.2015.266

**Published:** 2015-10-15

**Authors:** Z Wang, Y Zhou, X Hu, W Chen, X Lin, L Sun, X Xu, W Hong, T Wang

**Affiliations:** 1 School of Pharmaceutical Sciences, State Key Laboratory of Cellular Stress Biology, Xiamen University, Xiamen, Fujian, China; 2 Institute of Molecular and Cell Biology, A*STAR (Agency for Science, Technology and Research), Singapore

## Abstract

RILP (Rab7-interacting lysosomal protein) is a key regulator for late endosomal/lysosomal trafficking, and probably a tumor suppressor in prostate cancer. However, the role of RILP in other cancers and the underlying mechanism for RILP in regulating the invasion of cancer cells remain to be investigated. In this study, we showed that overexpression of RILP in breast cancer cells inhibits the migration and invasion, whereas the depletion of RILP by RNAi-mediated knockdown promotes the migration and invasion. We identified RalGDS (Ral guanine nucleotide dissociation stimulator) as a novel interacting partner for RILP, and truncation analysis revealed the N-terminal region of RILP is responsible for interacting with the guanine nucleotide exchange factor (GEF) domain of RalGDS. Immunofluorescence microscopy revealed that RalGDS can be recruited to the late endosomal compartments by RILP. Further investigations indicated that the overexpression of RILP inhibits the activity of RalA, a downstream target of RalGDS. Our data suggest that RILP suppresses the invasion of breast cancer cells by interacting with RalGDS to inhibit its GEF activity for RalA.

Diverse alternations of oncogenic factors can either activate or inactivate signaling pathways involved in cell proliferation, migration and apoptosis that are intimately associated with cancer development.^1, 2, 3^ Recent studies suggest that the derailed membrane trafficking is also closely related to cancer development. Activation or attenuation of signal transduction is usually linked to membrane trafficking. The recycling and degradation of surface receptors, such as EGFR, will influence downstream signaling pathways.^4, 5^ Therefore, the cross-talk between membrane trafficking and signaling pathway could be the novel mechanism associated with cancer development.

Alternations of the membrane trafficking machineries are established as the causes for some cancers. For examples, Rab25 is overexpressed in breast and ovary caners,^6^ and recent investigations suggest that Rab25 is also related to other cancers.^7, 8, 9^ Arf6 is a vital regulator for the invasive activity of breast cancer cells.^10^ Disordered membrane trafficking is emerging as an important property during tumorigenesis, thus the membrane trafficking machineries are potential therapeutic targets for cancer treatment.

Rab small GTPases are considered as the master regulators for membrane trafficking.^11^ The interactions between Rab proteins and their downstream effectors are involved in various steps of vesicle trafficking such as tethering and fusion. Aberrant activities of Rab proteins are closely related to some cancers.^12, 13, 14, 15^ Some Rab proteins mediate the trafficking of cargos, especially membrane proteins on the plasma membrane, such as integrin and E-cadherin. Their aberrant trafficking is proposed to be the underlying mechanism for the functional regulation of Rab protein in cancer cells.^16, 17^

Rab7, together with its downstream effector RILP (Rab7-interacting lysosomal protein), are the key regulators for late endosomal/lysosomal trafficking. RILP interacts with activated GTP-bound Rab7 through its carboxylic terminal region, whereas interacting with dynein/dynactin complex is mediated through its amino region, driving late endosomal/lysosomal trafficking, especially lysosomal positioning.^18, 19^ Rab7 has been demonstrated to be an important factor for cell growth and survival.^20, 21^ Recently, Steffan *et al.*^22^ found that RILP suppresses the invasion of prostate cancer cells through inhibiting the anterograde trafficking of lysosomes.^23^ Whether the potential role of Rab7-RILP in cell migration/invasion is also implicated in other cancers is of interest to investigate and the underlying molecular mechanism is yet to be defined.

In this study, we found that RILP suppresses the proliferation, migration and invasion of breast cancer cells. We also identified (Ral guanine nucleotide dissociation stimulator (RalGDS) as a novel interacting partner for RILP. The interaction of RILP with RalGDS modulates the activity of RalA. Our results suggest that RILP suppresses the invasion of breast cancer cells by modulating the activity of RalA through interaction with RalGDS.

## Results

### Overexpression of RILP inhibits proliferation, migration and invasion of breast cancer cells

**Figure 1:**
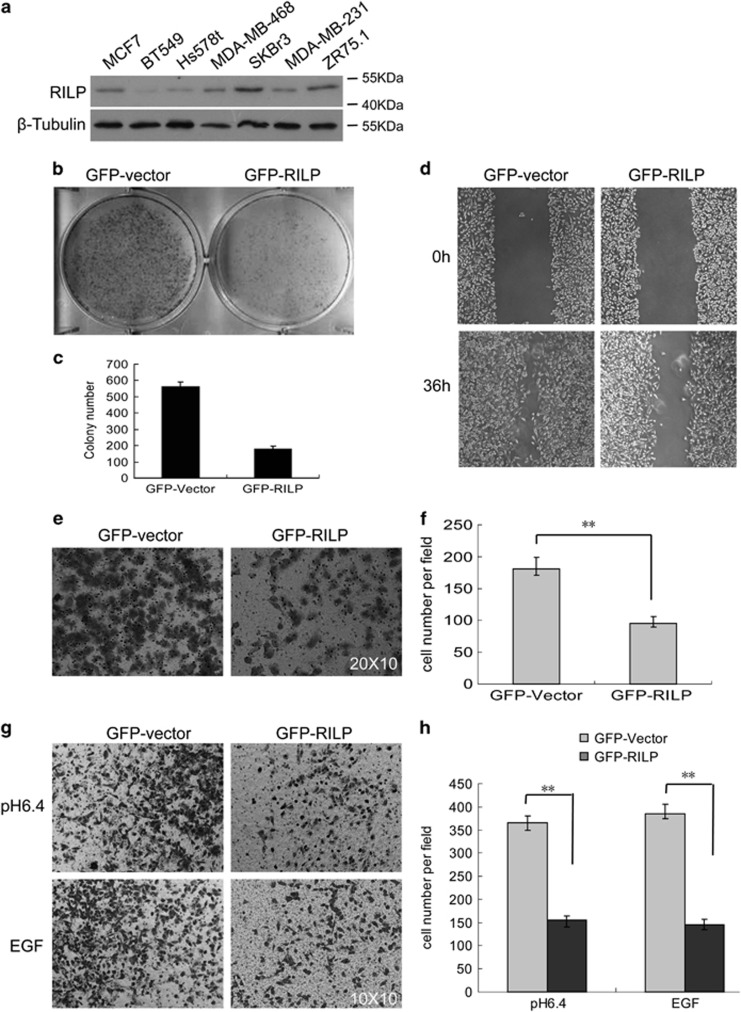
Overexpression of RILP Inhibits proliferation, migration and invasion. (**a**) Lysates of different breast cancer cell lines were subjected for western blot to analyze the protein level of RILP, indicating that RILP is expressed at lower levels in high invasive breast cancer cells (BT549, Hs578t and MDA-MB231) as compared with non-invasive cells such as MCF7 and ZR75.1. (**b**) MDA-MB-231 cells (1.0 × 10^4^) expressing EGFP vector or EGFP-RILP were grown in six-well plates for 4 days, then fixed and stained with 0.1% crystal violet. (**c**) Quantitative analysis of the colony number, showing that overexpression of RILP significantly inhibits cell proliferation. **P<0.01, *n*=3. (**d**) Wound-healing assay for MDA-MB-231 cells expressing EGFP or EGFP-RILP. The healing of wounds was imaged at 0 and 36 h, showing that overexpression of RILP inhibits migration. (**e**) Transwell migration assay. MDA-MB-231 cells (1.0 × 10^5^) expressing EGFP or EGFP-RILP were grown in upper chamber for 24 h. Cells migrated to the bottom side of the membrane of upper chamber were fixed, stained and imaged under microscope. Amplification: 20 × 10. (**f**) Quantitative analysis of the cell number in transwell migration assay from five random fields under microscope, showing overexpression of RILP significantly inhibits cell migration. **P<0.01, *n*=3. (**g**) Matrigel invasion assay. Transwell inserts were coated with growth factor-reduced Matrigel. MDA-MB-231 cells (1.0 × 10^5^) expressing EGFP or EGFP-RILP were grown with acidic media (pH6.4) or EGF-containing media for 24 h. The invaded cells were fixed, stained and imaged under microscope. Amplification: 10 × 10. (**h**) Quantitative analysis of the cell number in invasion migration assay from five random fields under microscope, showing overexpression of RILP significantly inhibits cell invasion. **P<0.01, *n*=3

RILP was recently shown to inhibit invasion of prostate cancer cells, but the underlying molecular basis remains elusive.^22, 23^ The role of RILP in breast cancer cells was investigated here. We examined the expression of RILP in different breast cancer cell lines, and found that the expression of RILP is lower in the highly invasive cells, such as BT549, Hs578t and MDA-MB-231 compared with less-invasive cell lines such as MCF7, SKBR3 and ZR75.1 (Figure 1a), suggesting downregulation of RILP is potentially associated with the increased invasion of breast cancer cells. To investigate this possibility, MDA-MB-231 cells were transfected with GFP-RILP, and G418 selection was used to generate cells stably expressing GFP-RILP. These cells were then examined for the effects of overexpressing RILP on the proliferation, migration and invasion.

To study the effects of RILP on cell proliferation, 1.0 × 10^4^ of the transfected cells expressing either GFP or GFP-RILP were seeded in six-well plate, and the cells were fixed after 4 days of growth. As shown in Figure 1b, overexpression of RILP significantly inhibited the cell proliferation, as the colony (>10 cells) number was markedly reduced compared with the control transfected cells expressing GFP (Figure 1c).

In wound-healing migration assay, the transfected cells were grown to confluence, and streaked by a yellow tip. The wound area was monitored. After 36 h, the wound area was almost recovered in control cells expressing GFP. However, the migration of cells expressing GFP-RILP was obviously delayed under similar condition (Figure 1d). The migration ability was also examined by the Transwell assay, As shown in Figure 1e and quantified in Figure 1f, the number of cells expressing GFP-RILP traversed the membrane is about two fold less than the control cells expressing GFP. These data suggest that overexpression of RILP inhibits the migration of breast cancer cells.

Transwell matrigel assay was used to investigate the invasion of breast cancer cells. Cells invaded into the lower chamber were fixed, and the average cell number was counted under microscope from 10 fields of 20 × 10 amplification. For cells overexpressing GFP-RILP, the invaded cells were significantly decreased compared with the control cells under acidic extracellular pH or EGF treatment (Figure 1g), both conditions supposed to promote invasion. The number of invaded cells expressing GFP-RILP is ~2.5-fold less than that of control cells (Figure 1h).

### shRNA-mediated knockdown of RILP promotes the proliferation, migration and invasion of breast cancer cells

**Figure 2:**
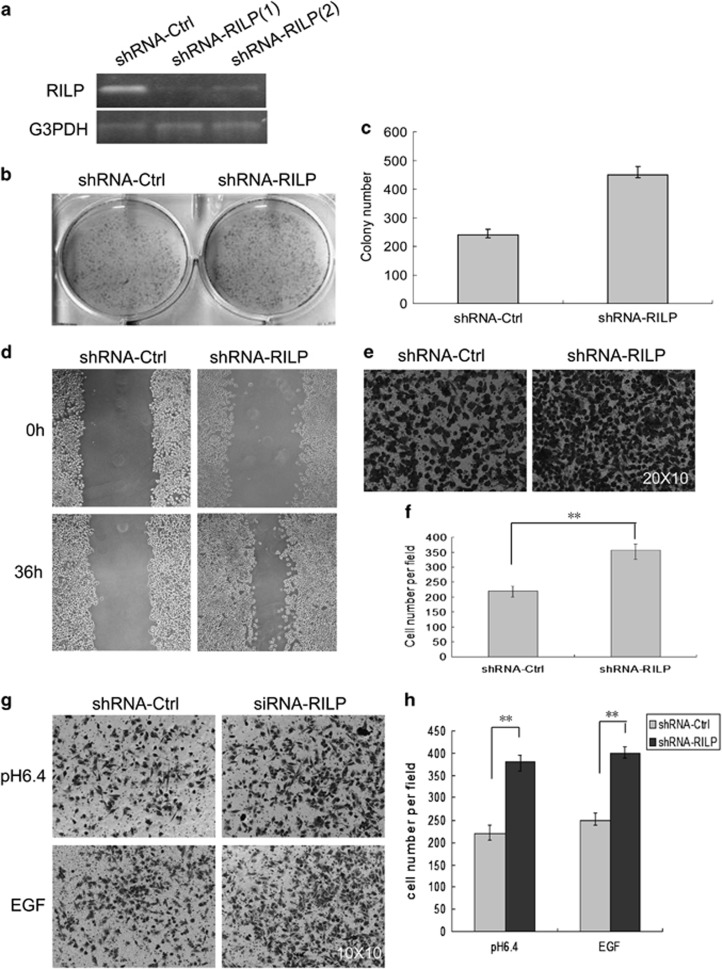
Depletion of RILP promotes the proliferation, migration and invasion. (**a**) MDA-MB-231 cells were transfected twice with pSuper-GFP-shRNA indicated, transfection efficiency (≥80%). Knockdown efficiency was examined by RT-PCR, showing shRNA-RILP(1) more efficiently suppressed the expression of RILP. (**b**) MDA-MB-231 cells (1.0 × 10^4^) expressing pSuper.GFP-shRNA-Ctrl or pSuper.GFP-shRNA- RILP were grown in six-well plates for 4 days, then fixed and stained with 0.1% crystal violet. (**c**) Quantitative analysis of the colony number, showing RILP depletion significantly promotes cell proliferation. **P<0.01, *n*=3. (**d**) Wound-healing assay for MDA-MB-231 cells expressing the constructs mentioned above. The healing of wounds was imaged at 0 and 36 h, showing RNAi of RILP enhanced cell migration. (**e**) Transwell migration assay. MDA-MB-231 cells (1.0 × 10^5^) expressing pSuper.GFP-shRNA-Ctrl or pSuper.GFP-shRNA-RILP were grown in upper chamber for 24 h. Cells migrated to the bottom side of the membrane of upper chamber were fixed, stained and imaged under microscope. Amplification: 20 × 10. (**f**). Quantitative analysis of the cell number in Transwell migration assay from five random fields under microscope, showing RILP depletion significantly enhanced cell migration. **P<0.01, *n*=3. (**g**) Matrigel invasion assay. Transwell inserts were coated with growth factor-reduced Matrigel. MDA-MB-231 cells (1.0 × 10^5^) expressing pSuper.GFP-shRNA-Ctrl or pSuper.GFP-shRNA-RILP were grown with acidic media (pH6.4) or EGF-containing media for 24 h. The invaded cells were fixed, stained and imaged under microscope. Amplification: 10 × 10. (**h**) Quantitative analysis of the cell number in invasion migration assay from five random fields under microscope, showing RILP depletion significantly enhanced cell invasion. **P<0.01, *n*=3

As indicated above, overexpression of RILP inhibits proliferation, migration and invasion. To independently validate its role, depletion of RILP by RNAi should have opposite consequence. We have used pSuper-mediated expression of shRNA to stably deplete RILP in MCF7 cells, which are considered as less-invasive breast cancer cells. As shown in Figure 2a, two cell pools expressing two independent shRNA for RILP were tested for the knockdown efficiency by RT-PCR. shRNA-RILP(1) has the better efficiency on suppressing the expression of RILP. Thus, the cells expressing shRNA-RILP(1) were used in the subsequent experiments.

The same approaches as described above were used to examine the effects of depletion of RILP on the proliferation, migration and invasion of MCF7 cells. As demonstrated in Figure 2b, cells expressing shRNA-RILP have enhanced proliferation, with the colony number of cells increased about two fold compared with that of the control cells expressing shRNA-ctrl (Figure 2c). Both wound-healing assay and Transwell assay revealed that the migration ability of MCF7 cells was enhanced significantly upon RILP depletion (Figures 2d and e). The migrated cells in Transwell assay were increased about twofold upon RILP depletion (Figure 2f). MCF7 has lower invasive ability under acidic pH or EGF treatment, however, the invasive ability was clearly enhanced when RILP was depleted (Figures 2g and h). The cells expressing shRNA-RILP invaded into the lower chamber were three fold more than control cells (Figure 2h). These data, together with the results from the above section, suggest that RILP plays a negative role in the proliferation, migration and invasion of breast cancer cells.

### RILP interacts with RalGDS

**Figure 3:**
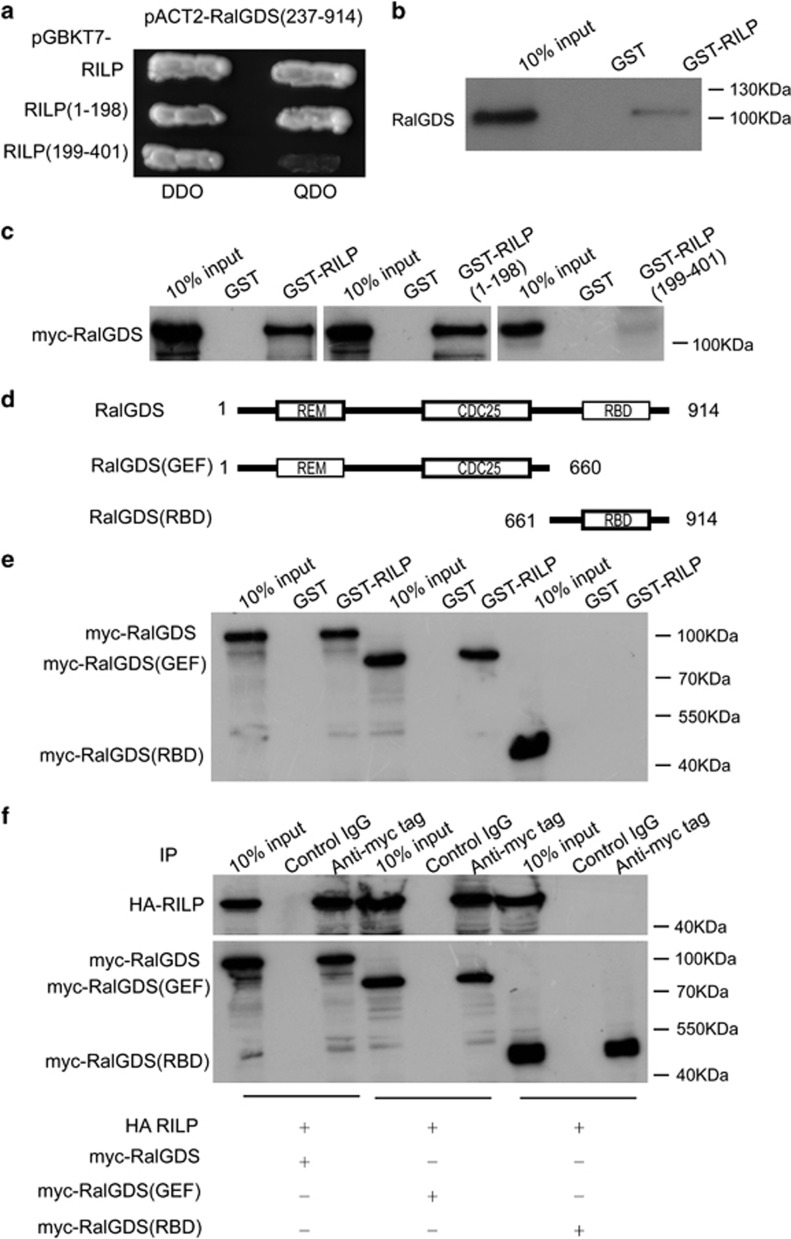
RILP interacts with RalGDS. (**a**) AH109 yeast cells expressing pGBKT7-RILP, pGBKT7-RILP(1–198) or pGBKT7-RILP(199–401) was mated with Y187 yeast cells expressing pACT2-RalGDS(237–914), respectively. Growth on DDO (-Leu/-Trp) media indicated diploids expressing both plasmids. Growth on QDO (-Leu/-Trp/-His/-Ade) media indicated positive interaction between two hybrid proteins. (**b**) The endogenous RalGDS in MCF cell lysates binds to GST-RILP fusion protein as revealed by GST-pulldown using immobilized GST-RILP. (**c**) MCF7 cells were transfected with myc-RalGDS, and the resulted cell lysates were subjected for GST-pulldown assays using GST-RILP, GST-RILP(1–198) or GST-RILP(199–401), respectively. The results showed that RalGDS binds to full-length RILP and N-terminal (1–198) but not C-terminal (199–401) region of RILP. (**d**) A diagram indicates the domain arrangement of RalGDS and the various truncation constructs. (**e**) MCF7 cells were transfected with myc-RalGDS, myc-RalGDS(GEF) or myc-RalGDS(RBD), respectively. The resulted cell lysates were subjected for GST-pulldown assays using GST-RILP. The results demonstrated that RILP interacts with the GEF domain of RalGDS. (**f**) MCF7 cells were co-transfected with HA-RILP and myc-RalGDS, myc-RalGDS(GEF) or myc-RalGDS(RBD), respectively. The resulted cell lysates were subjected for co-immunoprecipitation assays using rabbit anti-myc tag antibody, the immuno-complexes were revealed by western blot using anti-HA tag or anti-myc tag antibodies (9E10). The results confirmed that RILP interacts with the GEF domain of RalGDS

To uncover the molecular mechanisms underlying the role of RILP in regulating cancer cell properties, we searched for the interacting partners of RILP through yeast-two-hybrid screening using a pretransformed human fetal brain cDNA library. Screening at the highest stringency revealed nine independent clones that are positive for interaction with RILP. DNA sequencing revealed that two of the clones contain partial cDNA encoding RalGDS (residues 237–904 aa). The interaction between RILP and RalGDS(23-7914) was confirmed by yeast-two-hybrid system (Figure 3a). Interestingly, RalGDS only interacts with the N-terminal region (1–198 aa) of RILP, but not the C-terminal region (199–401 aa) of RILP. In addition, GST-pulldown assay showed that endogenous RalGDS in MCF7 cell lysates was retained by GST-RILP *in vitro* (Figure 3b). To confirm the interaction between RILP and RalGDS, myc-RalGDS in full length was expressed in MCF7 cells, and the cell lysates were subjected to GST-pulldown assay using GST-RILP, GST-RILP(1–198) and GST-RILP(199–401) fusion protein, respectively. The results again verified that RILP and its N-terminal but not C-terminal region interacts with RalGDS (Figure 3c).

Structurally, RalGDS contains two functional domains, guanine nucleotide exchange factor (GEF) domain at the N-terminal part and Ras-binding domain (RBD). The GEF domain consists of REM and CDC25 homolog regions (Figure 3d). To determine which region in RalGDS interacts with RILP, myc-tagged RalGDS, RalGDS(GEF) (truncated form containing GEF domain, 1–660aa) and RalGDS (RBD) truncated form containing RBD domain, 661–914 aa) were expressed in MCF7 cells, respectively. The resulted cell lysates were subjected to GST-pulldown assay using GST-RILP fusion protein. Western blot analysis revealed that RalGDS in full length and RalGDS(1–660) interacted with RILP efficiently (Figure 3e). To further confirm this interaction, HA-tagged RILP was co-expressed with myc-RalGDS, RalGDS(GEF) and RalGDS(RBD), respectively in MCF7 cells. The resulted cell lysates were processed for co-immunoprecipitation experiments. The results confirmed that GEF domain, not RBD domain in RalGDS is responsible for the interaction with RILP (Figure 3f). As the clones recovered from yeast-two-hybrid screening do not contain region encoding the N-terminal portion (1–236 aa), indicating that the N-terminal region is not essential for interaction and that the region consisting of residue 237–660 aa with CDC25 domain is likely responsible for interaction with RILP. Therefore, RalGDS is a novel interacting partner for RILP. As RalGDS functions as Ras-dependent GEF for Ral small GTPases,^24^ and is an important regulator in cancer behavior,^25^ the interaction of RILP with RalGDS is probably the mechanism (or part of the mechanism) for RILP regulating the proliferation, migration and invasion of breast cancer cells.

### RILP recruits RalGDS to late endosomal compartments

**Figure 4:**
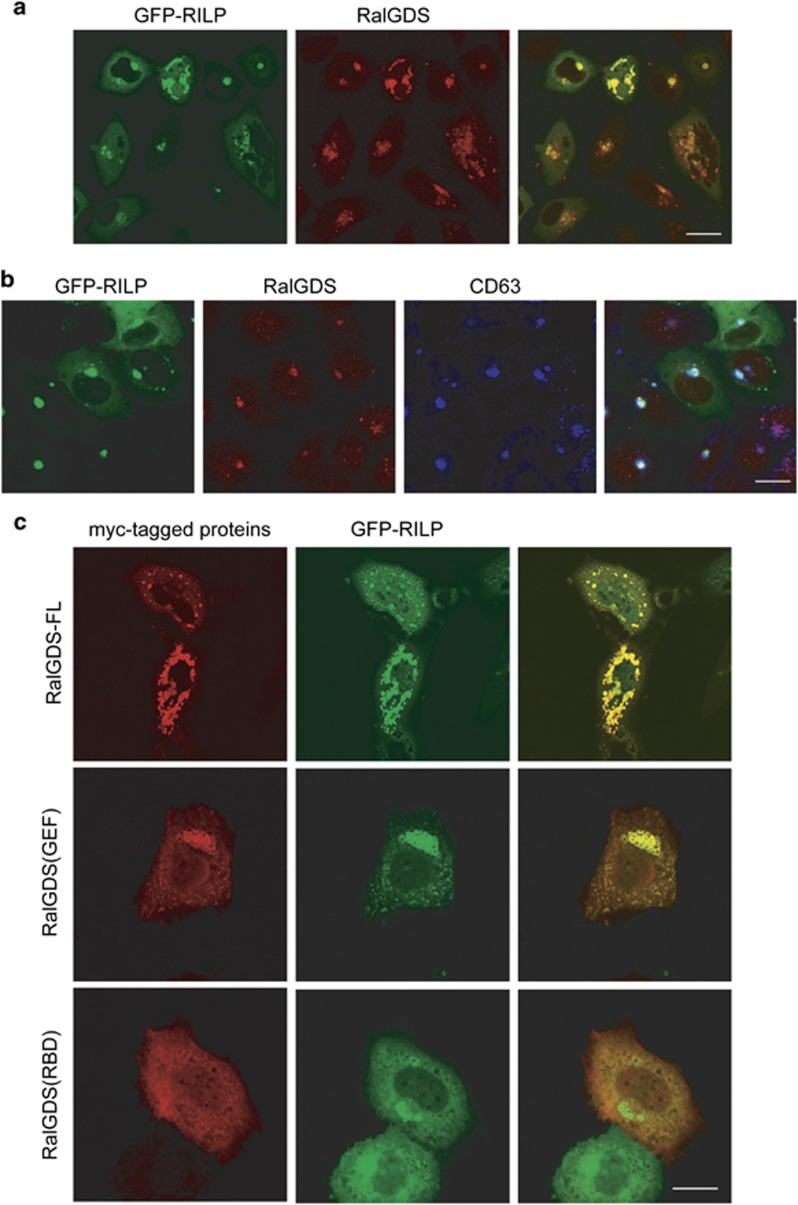
RILP recruits RalGDS onto the late endosome/lysosome. (**a**) MCF7 cells transfected with GFP-RILP were processed for immuno-labeling with anti-RalGDS antibody, followed by Texas-Red-conjugated secondary antibody, immunofluorescence confocal microscopy showed that endogenous RalGDS was recruited to the GFP-RILP marked compartments. Bar=20 *μ*m. (**b**) MCF7 cells transfected with GFP-RILP, and immuno-labeled with anti-RalGDS antibody and anti-CD63 antibody, RalGDS was revealed by Texas-Red conjugated secondary antibody, CD63 was revealed by Cy5-conjugated secondary antibody using confocal microscope, showing RILP recruits RalGDS to the CD63-labeled late endosomal/lysosomal compartments. Bar=20 *μ*m. (**c**) MCF7 cells were co-transfected with GFP-RILP and myc-RalGDS, myc-RalGDS(GEF) or myc-RalGDS(RBD), respectively. Cells were immuno-labeled with anti-myc tag antibody. Immunofluorescence confocal microscopy demonstrated that RalGDS-FL (full length) and RalGDS(GEF) but not RalGSD(RBD) was recruited by GFP-RILP. Bar=20 *μ*m

It has been well established that RILP associates with late endosomes/lysosomes and induces late endo-lysosomal compartments clustering into the peri-Golgi region.^18^ As RILP interacts with RalGDS, we wonder if RILP will affect the spatial distribution of RalGDS. Using immunofluorescence microscopy, it was observed that the endogenous RalGDS was recruited to the clustered structures marked by the overexpressed GFP-RILP (Figure 4a). These clustered structures were characterized as the late endosomal compartments marked by late endosomal membrane protein CD63 (Figure 4b). To further verify the recruitment of RalGDS by RILP, RILP was co-transfected with myc-RalGDS, myc-RalGDS(GEF) and myc-RalGDS(RBD) in MCF7 cells, respectively. As expected, singly expressed myc-RalGDS and its truncated forms distributed in cytoplasm (data not shown), however, when co-transfected with GFP-RILP, myc-RalGDS was recruited to the RILP-labeled structures and co-localized with GFP-RILP well, indicating that RILP recruits both endogenous and exogenous RalGDS in breast cancer cells. In addition, myc-RalGDS(GEF) but not myc-RalGDS(RBD) was shown to be recruited by RILP (Figure 4c), suggesting that the recruitment of RalGDS is dependent of the interaction of RalGDS with RILP.

### RILP inhibits the activation of RalA

**Figure 5:**
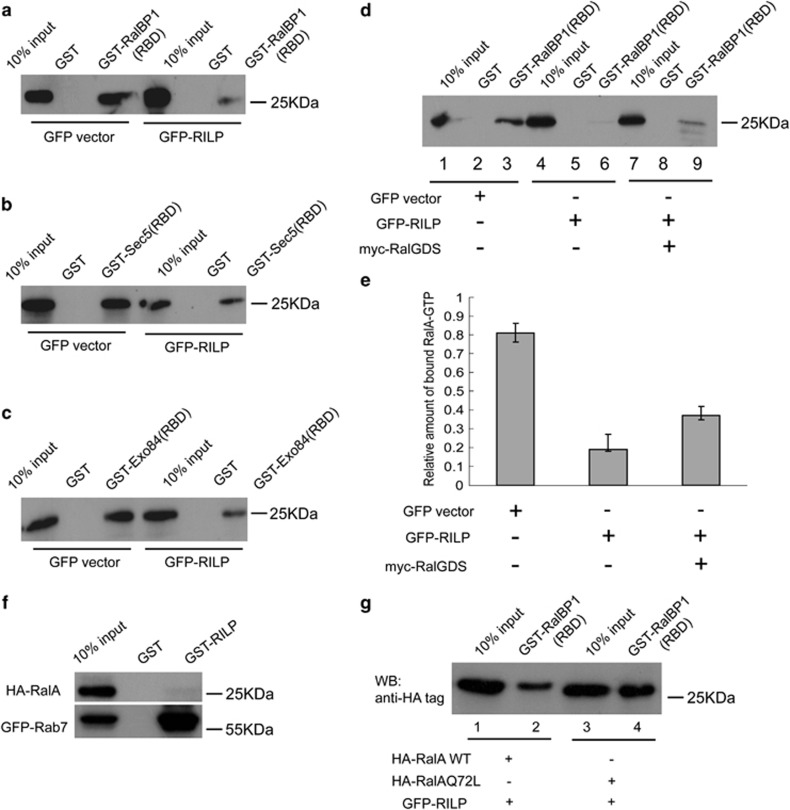
Effect of RILP on the activity of RalA. (**a**) MCF cells were transfected with control vector or vector for expressing GFP-RILP. Cells were lysed and the lysates were subjected for GST-pulldown assay using GST-RalBP1(RBD). The RalA bound by GST-RalBP1(RBD) was detected by western blot using anti-RalA antibody and is indicative of the amount of active RalA. (**b**) MCF cells were transfected with control vector or vector for expressing GFP-RILP. Cells were lysed and the lysates were subjected for GST-pulldown assay using GST-Sec5(RBD). The RalA bound by GST-Sec5(RBD) was detected by western blot using anti-RalA antibody and is similarly a reflection of active RalA. (**c**) MCF cells were transfected with control vector or vector for expressing GFP-RILP. Cells were lysed and the lysates were subjected for GST-pulldown assay using GST-Exo84(RBD). The bound RalA by GST-Exo84(RBD) was detected by western blot using anti-RalA antibody. (**d**) MDA-MB-231 cells were transfected with control vector or vector for expressing GFP-RILP, or vectors for co-expressing GFP-RILP and myc-RalGDS. Cells were lysed and the lysates were subjected for GST-pulldown assay using GST-RalBP1(RBD). The RalA bound by GST-RalBP1(RBD) was detected by western blot using anti-RalA antibody. (**e**) Quantitative analysis of the results from **d**, demonstrating RalA-GTP was significantly reduced upon overexpression of RILP, but partially restored when RalGDS was co-expressed with RILP. (**f**) GST-pulldown experiments showing RalA did not bind to GST-RILP. (**g**) MCF cells were double-transfected with GFP-RILP and HA-RalA WT, or GFP-RILP and RalAQ72L. The resulting lysates were subjected for GST-pulldown assay using GST-RalBP1(RBD). The RalA bound by GST-RalBP1(RBD) was detected by western blot using anti-HA tag antibody, showing RILP cannot inhibit the activity of RalAQ72L

RalGDS is the downstream effector of Ras and stimulates the GDP/GTP exchange of Ral small GTPases.^24, 25^ The active GTP-bound Ral can specifically interact with a distinct set of downstream effector proteins such as Ral-binding protein 1 (RalBP1), Sec5 and Exo84.^26, 27, 28, 29^ To investigate the functional implication of the interaction between RILP and RalGDS, the effect of RILP on the RalGDS-mediated activation of RalA was assessed by GST-pulldown assay using GST-RalBP1(RBD), GST-Sec5(RBD) or GST-Exo84(RBD) to measure the amount of the activated RalA-GTP from MCF7 cell lysates. The cell lysates resulted from MCF7 cells expressing GFP or GFP-RILP were subjected for GST-pulldown, Western blot experiments using RalA antibody revealed that GST-RalBP1(RBD), GST-Sec5(RBD) or GST-Exo84(RBD) can efficiently precipitate RalA from the cell lysates containing GFP, however, the amount of RalA bound to GST-RalBP1(RBD), GST-Sec5 (RBD) or GST-Exo84(RBD) was markedly decreased in cell lysates containing GFP-RILP (Figures 5a–c), indicating that overexpression of RILP reduced the amount of GTP-bound RalA.

To further examine the consequence of the interaction of RILP with RalGDS on the activation of RalA, we co-transfected GFP-RILP with myc-RalGDS in MDA-MB-231 cells, and the resulted cell lysates were processed for GST-pulldown experiments to detect the amount of RalA-GTP bound to GST-RalBP1(RBD). As shown in Figures 5d and e, the amount of Ral-GTP was significantly decreased when only GFP-RILP was expressed (Lane 4–6) and compared with cells expressing control GFP (lanes 1–3). However, when RILP was co-expressed with RalGDS, the amount of bound Ral-GTP was less affected, indicating overexpression of RalGDS can somewhat partially restore the activation of RalA. Additional GST-pulldown experiments revealed that RILP does not interact with RalA directly (Figure 5f). Taken together, RILP may inhibit the activation of RalA through the interaction with RalGDS, and consequently inhibiting the GEF activity of RalGDS.

The above results suggest that RILP is able to negatively regulate the GEF function of RalGDS, we next examined whether RILP influences the activation of the constitutively active mutant RalAQ72L. MCF7 cells were co-transfected with GFP-RILP and HA-RalAwt (wild type) or co-transfected with GFP-RILP and HA-RalQ72L, the resulted cell lysates were processed for GST-pulldown assay using GST-RalBP1(RBD), respectively. As shown in Figure 5g, the activated RalAwt is much less than the activated RalQ72L. As the mutant RalAQ72L is constitutively in a GTP-bound form in the cells, not mediated by GEF, the results again suggested that RILP inhibits the activation of RalA by disrupting the function of GEF in RalGDS.

### RILP inhibits invasion of breast cancer cells by inactivating RalA

**Figure 6:**
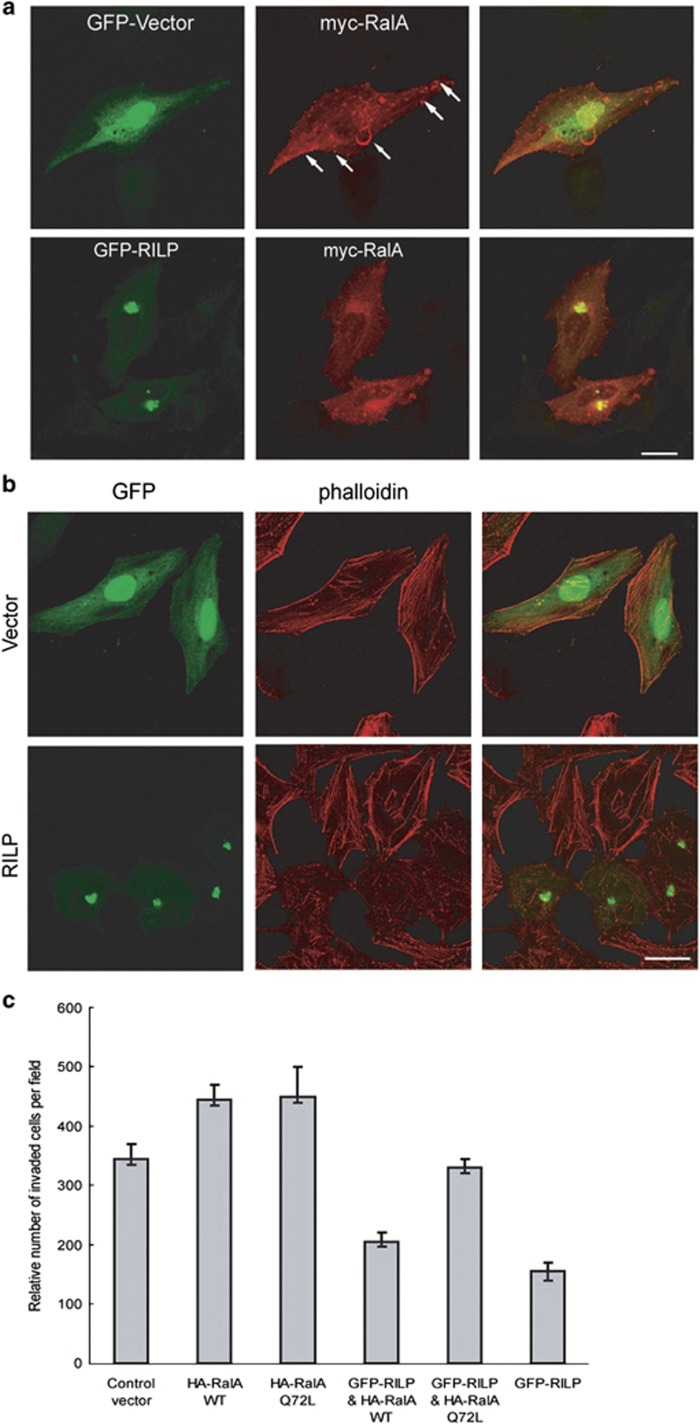
RILP inhibits the invasion of breast cancer cells by inactivating RalA. (**a**) MDA-MB-231 cells were transfected with HA-RalA and EGFP vector or EGFP-RILP, then processed for immunofluorescence confocal microscopy. RalA was associated with the plasma membrane and enriched in ruffles (upper panels, arrow), RILP disrupted the plasma membrane association of RalA (lower panels), instead, inducing clustering of RalA onto the peri-Golgi region. Bar=20 *μ*m. (**b**) RILP induces re-arrangement of actin cytoskeleton. MCF7 cells were transfected with EGFP vector (upper panels) or EGFP-RILP (lower panels), and stained with Rhodamine-conjugated phalloidin to reveal actin cytoskeleton, showing the overexpression of RILP decreased actin stress fibers and cortical actin. Bar=20 *μ*m. (**c**) MDA-MB-231 cells were transfected with control vector, HA-RalA WT, HA-RalAQ72L or GFP-RILP, respectively, or co-transfected with GFP-RILP and HA-RalA WT or GFP-RILP and HA-RalAQ72L. The cells were processed for Matrigel invasion assay. The invaded cells were fixed, stained and imaged under microscope. Quantitative analysis of the cell number in invasion migration assay from five random fields under microscope showed that RalAQ72L counteracted the inhibition effects on the invasion

To further investigate the effects of RILP on RalA, myc-RalA was co-expressed with GFP-RILP or GFP-Vector in MDA-MB-231 cells. Immunofluorescence microscopy revealed that RalA usually located at the leading edge and membrane ruffle structures (Figure 6a, upper panels). However, when co-expressed with RILP, the distribution of RalA was markedly altered, with some of RalA accumulated at the nuclear region and RalA associated with membrane ruffle was reduced significantly (Figure 6a, lower panels), indicating that RILP disrupts the translocation of RalA to the plasma membrane, which is essential to simulate the cell motility.

RalA regulates actin cytoskeleton organization by mediating the activity of downstream factor Rho/CDC42.^30, 31, 32^ We examined whether inhibition of RalA by overexpressing RILP affects the arrangement of actin filaments. It was observed that overexpression of RILP resulted in the decrease of stress actin fibers (which are supposed to provide contractile force for cell migration) and cortical actin in MDA-MB-231 cells, without significant alternation in vector-transfected cells (Figure 6b). These observations suggest that RILP may influence RalA signaling pathway to induce re-arrangements of actin cytoskeleton.

To test whether RILP inhibits the invasion of breast cancer cells through inactivating RalA, GFP-RILP was co-expressed with HA-RalAwt (wild type) or HA-RalAQ72L constitutively active mutant in MDA-MB-231 cells, the cells were processed for invasion assay. As expected, RalA promotes invasion of breast cancer cells. The statistics analysis revealed that RILP inhibits the invasion compared with the control vector. However, RalAQ72L mutant can counteract the inhibition effects of RILP significantly, whereas RalA WT has no major effects (Figure 6c). As RalQ72L is the constitutively active mutant and its activity is less dependent of GEF, the results suggested that RILP cannot inhibit the activity of RalAQ72L, thus RalQ72L promotes the invasion; however, RILP can inactivate RalAwt, thus, RalAwt cannot counteract the inhibition effects caused by RILP. Taken together, RILP may inhibit the invasion of breast cancer cells by modulating the activity of RalA through the interaction with RalGDS to negatively regulate the GEF function.

### RILP negatively regulates ERK signaling pathway

**Figure 7:**
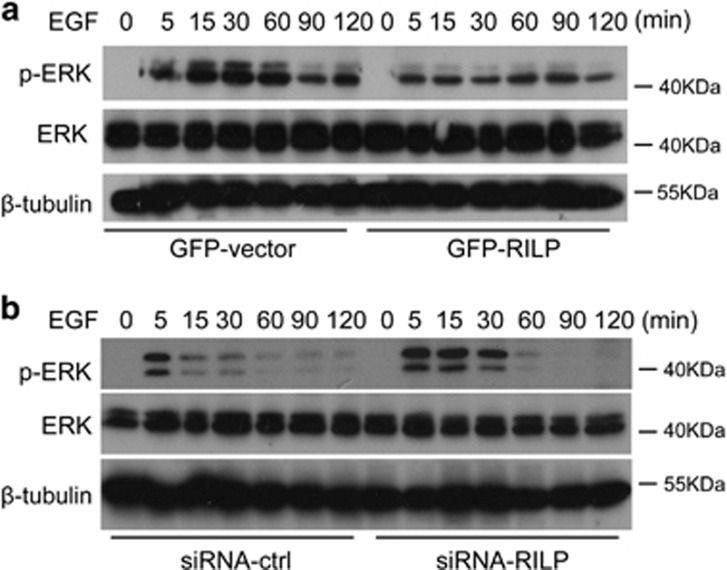
RILP negatively modulates ERK signaling pathway. (**a**) MDA-MB-231 cells stably expressing EGFP vector or EGFP-RILP were serum-starved for 18 h, then stimulated with EGF (10 ng/ml) for the indicated time. Cells were lysed and equal amounts of protein were subjected for western blot to detect total and phosphorylated ERK, showing overexpression of RILP inhibited noticeably the phosphorylation of ERK in response to EGF. (**b**) MDA-MB-231 expressing shRNA-Ctrl or shRNA-RILP were serum-starved for 18 h, then stimulated with EGF (10 ng/ml) for the indicated time. Cells were lysed and equal amounts of protein were subjected for western blot to detect total and phosphorylated ERK, showing RILP depletion enhanced the phosphorylation of ERK in response to EGF

ERK pathway is important for cell proliferation and tumorigenesis, and is regulated by Ras and Ral.^33, 34^ As RalGDS is the downstream effector of Ras and serves as GEF for Ral, the interaction of RalGDS with RILP may affect ERK signaling. To address this hypothesis, MDA-MB-231 cells overexpressing GFP-RILP or shRNA-RILP were starved and stimulated with EGF in a time course manner, and the phosphorylation of ERK was monitored by western blot using antibody specifically against phosphorylated ERK. It is demonstrated that overexpression of RILP significantly inhibits the phosphorylation of ERK (Figure 7a), whereas shRNA-mediated knockdown of RILP enhanced the phosphorylation of ERK (Figure 7b), suggesting that RILP negatively regulates ERK signaling pathway in breast cancer. Although Ras-RalGEF activates both PI3K and ERK pathway, our experiments demonstrated that RILP is involved in ERK pathway, but not affecting AKT pathway (data not show), consistent with the previous investigation.^23^

## Discussion

Lysosomal trafficking is emerging as novel therapeutic target for cancer.^35^ RILP interacts with Rab7, Rab34 and dynein/dynactin complex to regulate late endosomal/lysosomal trafficking from peripheral region to peri-Golgi region marked by MTOC,^18, 19, 36^ this event may inhibit the anterograde transport and result in the suppression of invasion of prostate cancer cells.^22, 23^ Our works demonstrated that RILP suppresses cell proliferation, migration and invasion of breast cancer cells, supporting that RILP may function as tumor suppressor. The downregulation of RILP in invasive breast cancer cells is consistent with this possibility.

In this study, we also characterized RalGDS as a novel interacting partner for RILP. RalGDS interacts with Ras/Ral through RBD at its C-terminal, this interaction is essential for the Ras-dependent activation of its guanine nucleotides exchange activity.^37^ The N-terminal part interacts with PDK1 and beta-arrestin, both interactions regulate the activation of Ral pathway.^38, 39^ We defined a region containing CDC25 domain at the N-terminal GEF domain of RalGDS responsible for the interaction with RILP. Therefore, the interaction between RILP and RalGDS may not affect the interaction of RalGDS with Ras/Ral, but interfere with the GDP/GTP exchange activity of RalGDS due to the binding to the central CDC25 region.

RalGDS interacts with activated H-Ras, R-Ras and Rap1 small GTPases.^37^ RalGDS serves as GEF for Ral proteins (RalA and RalB), coupling Ras to Ral signaling pathway.^24, 25^ RalGDS can activate RalA through Ras-dependent or independent manners.^37, 38, 39^ Activation of RalGDS promotes prostate cancer metastasis.^40^ Although RalA and RalB may have different functions, both are involved in proliferation, migration and invasion of cancer cells.^41^ We found that RILP interacts with RalGDS, and overexpression of RILP inhibits the activation of RalA, providing a novel mechanism for the roles of RILP in regulating proliferation, migration and invasion of breast cancer cells. As the interacting region is located at the CDC25 region of RalGDS, RILP may physically block the GEF activity toward Ral GTPases.

RalBP1 is a downstream effector of Ral GTPases, it functions as GTPase activation protein (GAP) for Rho/CDC42.^30^ Through interaction with RalBP1, Ral proteins mediate downstream effectors Rho/CDC42 to regulate the organization of actin cytoskeleton.^31^ Activated Ral is translocated from the cytoplasm to the plasma membrane, regulating the formation of filopodia and membrane ruffle, which facilitate cell migration.^32^ Inhibition of Ral decreases the actin fibers in cancer cells.^39^ Coincidentally, our observations demonstrated that overexpression of RILP altered the plasma membrane association of RalA, resulting its sequestering at the RILP-labeled region, the underlying mechanisms for this phenomenon is probably due to the interaction of RILP with RalGDS, which may consequently inhibit the activation of RalA and prevent the translocation of RalA to plasma membrane. In addition, overexpression of RILP reduced the actin stress fibers, one possible explanation for this observation is that RILP disrupts the interaction of RalA with RalBP1, but does not affect the interaction of RalBP1 with Rho/CDC42 through GAP domain, which activates the GTP hydrolysis of GTP-Rho/CDC42, resulting in rearrangement of actin cytoskeleton.

Interestingly, both RalGDS and RalA are involved in secretory-trafficking pathway. RalGDS was found to mediate exocytosis of Weibel–Palade bodies in endothelial cells.^42^ The previous investigations demonstrated that exocyst complex is an effector of Ral GTPase. Activated Ral can interact with Sec5 and Exo84 to mediate exocytosis.^27, 28, 29^ It was reported that RalA controls exocytosis of large dense core secretory granules,^43^ and also regulates exocytosis of insulin granules in pancreatic *β*-cells.^44^ Secretory trafficking delivers vesicles and cell contents targeting to the plasma membrane, important for diverse cellular events, such as epithelial polarity establishment, neurite growth,^45, 46^ and recent findings indicated that exocytosis regulates cell migration.^47, 48^ The overexpression of RILP induces clustering of RalGDS at MTOC region, at the same time, inhibits the activation of Ral, implicating RILP may inhibits cell migration and invasion through interfering Ral-mediated exocytosis of proteins to the leading edge during cell migration/invasion.

**Figure 8:**
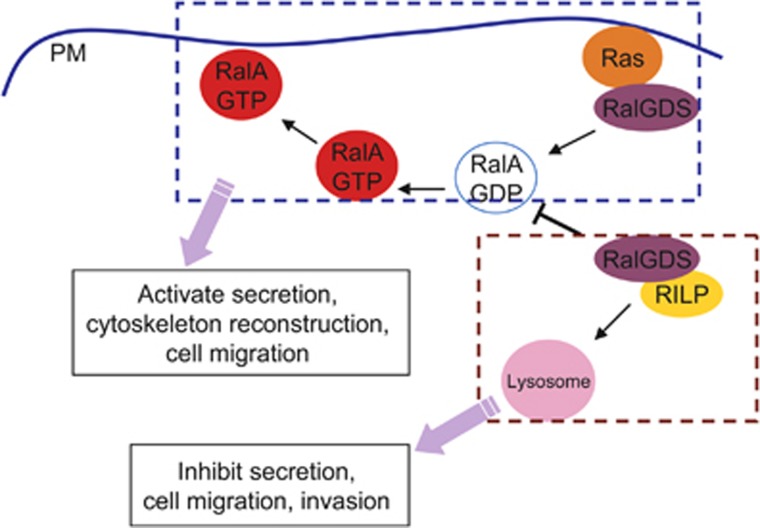
A working model depicting that RILP interacts with RalGDS to inhibit Ral pathway. Normally, RalGDS activates RalA through interaction with Ras, and translocate the activated RalA to the plasma membrane (PM), this activation is required for RalA's activity for secretion and cell migration. The interaction of RalGDS with RILP will inhibit the activation of RalA, probably due to blockage of GEF activity of RalGDS by RILP, this inhibition results in the sequestering of RalGDS and/or RalA, and subsequently inhibits the cell migration and invasion of cancer cells

Normally, RalGDS activates RalA through interaction with Ras, and translocate the activated RalA to the plasma membrane (Figure 8), this activation is required for RalA's activity during secretion and cell migration. The interaction of RalGDS with RILP will inhibit the activation of RalA, probably due to the blockage of GEF activity of RalGDS, this inhibition results in the sequestering of RalGDS and/or RalA (Figure 8). Together, we hypothesize that RILP interacts with RalGDS, and consequently inhibits its GEF activity, resulting in inhibition of RalA activation, thus suppresses the secretion and cytoskeleton reconstruction required for cell migration and invasion of cancer cells.

## Materials and Methods

### Expression constructs

cDNA encoding Myc-RalGDS in full length (1–914 aa), myc-RalGDS(GEF) (1–660aa) and myc-Ral (GDRBD) (661–914aa) were constructed by inserting the respective coding fragment, generated by standard PCR approach, into ECOR1/Not1 sites of PCIneo-myc vector. Coding region for RBD (396–520 aa) in RalBP1, RBD domain (1–120 aa) in Sec5 and RBD domain (122–333 aa) in Exo84 were retrieved by PCR from human fetal brain cDNA library (BD Biosciences, Palo, Alto, CA, USA) and cloned into EcoR I/Not I sites of pGEX-4 T-1 vector (GE Healthcare Life Sciences, Fairfield, CT, USA) to generate constructs for expression of GST-RalBP1(RBD), GST-Sec5(RBD) or GST-Exo84(RBD). Human RalA cDNA (wild type) was purchased from Upstate Biotechnology (Lake Placid, NY, USA), and subcloned into pDHA vector to generate HA-RalA. pACT2-RalGDS(237-914aa) was retrieved from the positive interaction yeast clone in yeast-two-hybrid screening. All constructs were confirmed by DNA sequencing.

cDNA for HA-RILP was generated by subcloned the coding fragment of RILP cDNA into pDHA vector. Constructs for GFP-RILP, GFP-Rab7, GST-RILP, GST-RILP(1-198) and GST-RILP(199-401) were described previously.^36^ pGBKT7-RILP, pGBKT7- RILP(1-198) and pGBKT7-RILP(200-401) were described.^49^

### Antibodies

The rabbit polyclonal antibodies against RalGDS or RILP were purchased from SANTA CRUZ (Delaware Avenue, CA, USA). The mouse monoclonal antibodies (mAb) against RalA and HA were from Upstate Biotechnology. The rabbit mAbs against phosphor-ERK (Thr202/Tyr204) and ERK were purchased from Cell Signaling Technology (Danvers, MA, USA). The mAb against human CD63 was obtained from the Developmental Studies Hybridoma Bank maintained by the University of Iowa (Department of Biological Science, Iowa City, IA, USA). The mouse mAb against GFP was from Clontech (Palo Alto, CA, USA). The mouse mAb against Myc (9E10) was obtained from American Type Culture Collection (ATCC, Manassas, VA, USA). The mouse mAb against *β*-tubulin was obtained from Sigma (St. Louis, MO, USA). Texas-Red conjugated secondary antibodies were from Jackson ImmunoResearch (West Grove, PA, USA). HRP-conjugated secondary antibodies were purchased from Pierce (Rockford, IL, USA).

### Cell culture and transfection

The human breast cancer cell lines MDA-MB-231 and MCF7 were from ATCC and grown in DMEM medium supplemented with 10% fetal bovine serum (Gibco, Ann Arbor, MI, USA) in a 5% CO_2_ incubator at 37 °C. Transfection experiments were carried out using lipofectamine 2000 transfection reagent (Thermo Fisher Scientific, Waltham, MA, USA) according to the manufacturer's protocol. To achieve higher transfection efficiency, cells were transfected twice and screened with G418 (Gibco).

### RNA interference

pSuper.GFP-vector mediated expression of shRNA was used to suppress the expression of RILP, using shRNA-RILP1 (5′-GCAGCGGAAGAAGATCAAGTT-3′) and shRNA-RILP2 (5′-GATCAAGGCCAAGATGTTATT-3′). The scramble oligonucleotides (5′-ACTTCGAGCGTGCATGGCTTT-3′) were used to generate control shRNA expression vector.

The knockdown efficiency was monitored by the examining the transcript of RILP assessed by RT-PCR. Total RNA was isolated with Trizol (Thermo Fisher Scientific) according to the manufacturer's protocol. cDNA was synthesized using the SuperScript First-Strand cDNA synthesis system (Thermo Fisher Scientific) according to manufacturer's instruction. PCR was performed using the specific primers (forward primer, 5′-GCAGCGGAAGAAGATCAAGGC-3′ and reverse primer, 5′-GACAAAGGTGTTCGTGGAGGG-3′) for RILP.

### Cell growth assay

Cells were seeded in six-well plate (1.0 × 10^4^ cells/well) and grown for 4 days. The cells were fixed with 4% paraformaldehyde and stained with 0.1% crystal violet. The cell colonies (≥10 cells) were counted under microscope.

### Cell migration assay

For wound-healing assay, cells were plated at a density of 1.0 × 10^5^ cells in a 35-mm plate. After 24 h, the cell monolayer was wounded with a pipette tip. The cell debris was washed out with PBS. Cells were grown for another 36 h, and the migration of the cells was examined using an inverted microscope.

For Transwell assay (24-well, Corning, Inc., Corning, NY, USA), cells were starved in DMEM containing 1.5% FBS for 16 h, then transferred to the upper chamber (1.0 × 10^5^ cells/chamber) with DMEM containing 10% FBS in the lower chamber. The chamber culture was incubated at 37 °C for 24 h. The non-migrated cells were washed off from the upper surface of the membrane. The migrated cells on the bottom surface were stained with 0.1% crystal violet and counted under microscope.

### Cell invasion assay

Cells invasion assay was done the same as Transwell migration assay, except that the transwell inserts were coated with growth factor-reduced Matrigel (BD Biosciences). Cells (1.0 × 10^5^) suspended in serum-free media were plated on top of the Matrigel. Chemoattractant media was titrated to pH6.4 or containing 100 ng/ml EGF. Cells were allowed to invade for 24 h. The cells were fixed in 4% paraformaldehyde for 20 min, and stained with 0.1% crystal violet for 30 min. The cells remaining on the top of the insert (those that did not invade) were removed with a cotton swab. The number of invaded cells was counted in five random fields for each insert under an inverted microscope.

### Yeast two-hybrid system

Yeast two-hybrid-screening was performed as described previously.^49^ In brief, the yeast strain AH109 harboring the pGBKT7 vector containing RILP fused to the Gal4 DNA-binding domain was mated with 6.05 × 10^7^ independent yeast colonies (strain Y187) pretransformed with a human fetal brain cDNA library fused to the Gal4 DNA activation domain in the pACT2 vector (BD Biosciences). Screening was done at the highest stringency on QDO selection media (-Trp/-Leu/-His/-Ade). To verify the interaction, Y187 strain was re-transformed with the plasmids recovered from the positive clones, and mated with AH109 strain expressing various RILP constructs. The diploids were selected on DDO (-Trp/-Leu) or QDO media.

### GST-pulldown and co-immunoprecipitation assay

MCF7 cells were transfected with the indicated plasmids, and lysed in lysis buffer (25 mM Tris-HCl containg 100 mM NaCl, 1.0% Triton X-100, 1.0 mM PMSF and proteinase inhibitor cocktail (Roche, Nutley, NJ, USA), pH 7.4). The cell lysates were clarified by spinning at 14 000 rpm for 30 min. The supernatants were used for GST-pulldown or Co-immunoprecipitation assay.

For GST-pulldown assay, GST-fusion proteins (20 *μ*g) immobilized onto the GST-Sepharose-4B resin (GE Healthcare Life Sciences) were used to bind the interacting proteins in the cell lysates. For co-immunoprecipitation, the clarified supernatants were incubated sequentially with anti-myc antibody for 2 h and then with proteinA/G agarose (Sigma-Aldrich, St. Louis, MO, USA) for overnight at 4 °C. After excessive washing, the bound proteins on GST-beads or proteinA/G agarose beads were assessed by western blot analysis.

### Western blot analysis

Proteins resolved by SDS-PAGE were transferred onto PVDF membrane. The membrane was blocked with 5% milk in TBST (20 mM Tris, 137 mM NaCl, 0.1% Tween 20, pH 7.5) and probed with appropriate primary antibodies. HRP-conjugated secondary antibodies and ECL-plus from GE Healthcare were used to visualize protein bands on Kodak BioMax XAR film. Densitometry analysis was performed using Image J software.

### Immunofluorescence microscopy

Immunofluorescence microscopy experiments were performed as described.^36^ In brief, cells grown on cover glasses were washed with PBSCM (PBS containing 1.0 mM CaCl2 and 1.0 mM MgCl2) and then fixed with 3% paraformaldehyde in PBSCM at 4 °C for 30 min. After washing with PBSCM supplemented with 50 mM NH_4_Cl, cells were permeabilized with 0.1% saponin (Sigma) in PBSCM for 15 min at room temperature, and were subjected for immuno-staining using the primary antibodies indicated, followed by Texas red-conjugated secondary antibodies. GFP-proteins were viewed directly under GFP channel in microscope. The immuno-labeled cells were analyzed with Carl Zeiss LSM5 EXITER laser scanning confocal microscope (Zeiss, Jena, Germany).

### Statistics analysis

Three independent experiments were carried out. GraphPad Software, Prism 5.0 was utilized to perform all statistics analysis. Two-tailed Mann–Whitney *U*-tests were performed to indicate statistical significance.

## Abbreviations

RILP: Rab7-interacting lysosomal protein
RalGDS: Ral guanine nucleotide dissociation stimulator
GEF: Guanine nucleotide exchange factor
RBD: Ras or Ral-binding domain
RalBP1: Ral-binding protein 1
MTOC: Microtubule organizing center
